# Geriatric Neuro-Oncology in the Middle East: A Sultanate of Oman Experience

**DOI:** 10.3390/neurolint13020024

**Published:** 2021-05-28

**Authors:** Omar Al-Taei, Abdulrahman Al-Mirza, Tariq Al-Saadi

**Affiliations:** 1 Sultan Qaboos University College of Medicine, Al-Khoudh, Muscat 123, Oman; s121283@student.squ.edu.om (O.A.-T.); s119253@student.squ.edu.om (A.A.-M.); 2 Department of Neurology & Neurosurgery—Montreal Neurological Institute, Faculty of Medicine, McGill University, Montreal, QC H3A 0G4, Canada; 3 Department of Neurosurgery, Khoula Hospital, Muscat 116, Oman

**Keywords:** brain tumors, malignancy, Meningiomas, Glioblastoma, elderly

## Abstract

Brain tumors are primary or metastatic malignancies of the central nervous system (CNS) with significant morbidity and mortality. The overall prevalence of cancer including brain cancer has increased by more than 10% according to the National Institute of cancer statistics. The average percent increase in primary brain tumor incidence for ages 75–79, 80–84, and 85 and older were 7%, 20.4%, and 23.4%, respectively. This manuscript describes a retrospective study of geriatric cases admitted to the Neurosurgical Department in Khoula Hospital (KH) and diagnosed with brain cancer from 1 January 2016 to 31 December 2019. Of the study cohort, 58.5% were more than 75 years of age. The male-to-female ratio was (1:1.1). Meningiomas are found to be the commonest tumor (52.8%) followed by glioblastoma (GBM) (18.9%). Most of the patients had a Glasgow coma scale (GCS) score of 14–15 on admission (69.9%). Patients diagnosed with a non-meningioma tumor had lower GCS score on admission compared to meningioma patients with statistical significance (*p* = 0.04). Also, there was a significant difference between the length of stay (LOS) and the type of intervention (surgical vs. conservative), in which patients received a conservative type of management found to have a shorter length of stay (LOS) compared with the patients who underwent surgical intervention (*p* < 0.005). In Oman, the number of geriatric oncology cases remained stable over the 4 years. The incidence of geriatric neuro-oncology cases was higher in patients aged more than 75 years of age. Finally, the GCS score was affected by the type of tumor. The length of stay varies according to the treatment administered. Special care must be taken when dealing with geriatric neuro-oncological cases due to the high potential rate of mortality and morbidity among those group, and a more holistic approach is recommended as an essential need to evaluate the overall situation of those patients and manage them accordingly.

## 1. Introduction

The most apparent explanation for the relationship between cancer and age is the time length of carcinogenesis [1]. Molecular aging is associated with changes that support and other changes that may oppose carcinogenesis. For example, pro-carcinogenesis changes include DNA adducts, DNA methylation, and genetic instability [2]. Brain tumors are primary or metastatic malignancies of the central nervous system (CNS) with significant morbidity and mortality. The overall prevalence of brain tumors is increasing with a noticeable increase in patients aged 60 and above [3]. Between 1973 and 1985 the average percent increase in primary brain tumor incidence for ages 75–79, 80–84, and 85 and older were 7%, 20.4%, and 23.4% respectively. However, since 1970 the incidence of primary brain tumors in people over the age of 70 has increased seven fold [3]. In the last three decades in the United States, there were major increases in mortality due to incidence rates of primary brain tumors increasing [4]. Annually in the United States, greater than 20,000 patients are diagnosed with a primary malignant glial neoplasm [5]. According to the ministry of health in Oman, local data found the incidence of astrocytic tumors is 53% followed by medulloblastoma 19% [6]. The prevalence rate of both malignant and primary CNS tumors is 18.16 per 100,000 persons for the general population. However, this incidence for all primary CNS tumors is highest among 75 to 84 years of age (63.75 per 100,000 persons) [7]. Aging is considered one of the primary risk factors for cancer with a cut-off age of 65 years and above accounting for 60% of newly diagnosed malignancies [8]. Many reports have highlighted that the cancer mortality rate for persons aged 65 years and above is approximately 16 times higher than those who are younger [8]. Moreover, there are certain histologies of tumor commonly seen in the elderly such as; the incidence of glioblastoma (GBM), anaplastic astrocytoma, and meningioma rises proportionally with age [7]. Meningioma is the commonest intracranial extra-axial tumor in the elderly [9]. The incidence of meningioma in the elderly is estimated to be about 30% of all intracranial tumors [10]. Usually, meningiomas are World Health Organization (WHO) grade 1, which are benign and have a slow growth rate [10]. Meningioma increases with age, as well as higher life expectancy and more frequent use of diagnostic imaging resulting in an increased diagnosis of meningioma [10]. On the other hand, GBM is the most common aggressive primary CNS tumor. The incidence of GBM increases tremendously with age peaking between ages of 65 to 84 [7]. The overall incidence of GBM in the elderly is 13.16 per 100,000 persons [11]. Patients with GBM often have a poor prognosis with a median survival rate of 2 years despite therapeutic advances [11]. Also, anaplastic astrocytoma (AA) is a diffusely infiltrating malignant, astrocytic primary brain tumor [12]. The incidence of AA is approximately 0.48 per 100,000 people/year [13]. Moreover, pituitary tumors are generally benign and slow-growing lesions that vary in clinical manifestation. There are approximately 7% of pituitary tumors in patients more than 65 years of age [14]. To the best of our knowledge, no local research has been conducted regarding geriatric neuro-oncology. This study aims to retrospectively analyze the prevalence and outcome of common brain tumors and malignancies in elderly patients at the Department of Neurosurgery in a tertiary hospital in Oman. Oman is considered to have one of the best well-rounded healthcare systems according to World Health Organization reports [15,16]. The Department of Neurosurgery in Khoula Hospital (KH) is the core neurosurgical center in the country with average admission of 1600 patients yearly [17,18]. In this study, we chose a cut of 65 years and older according to the local definition, taking into account the increase in life span during recent decades as well as the improvement in the quality of life.

## 2. Methods and Study Design

### 2.1. Study Group

This is a retrospective study conducted at KH located in Muscat, Sultanate of Oman. The study was approved by the Research Ethical Committee at Khoula Hospital/Ministry of Health (7 December 2020; ID:PRO122020072). Medical records of 106 patients who are above the age of 65 and admitted to the neurosurgical ward and diagnosed with a brain tumor or brain malignancy, from the period of 1 January 2016 to 31 December 2019 were included. The study includes both Omani and non-Omani patients. Patients with the following features are excluded: non-elderly patient (below 65 years), patients diagnosed with a tumor before the age of 65, patients admitted to the neurosurgical department for reasons other than tumor or malignancy, outside the study period (from 1 January 2016 to 31 December 2019), and patients with missing or incomplete data.

### 2.2. Data Collection

Data were obtained from the health information system (Al-Shifa Electronic Health Record System) and included: patient demographics (age, gender), presenting symptom, pre-operative medical conditions, post-operative complications, previous surgical history, preoperative and postoperative Glasgow coma scale (GCS), site of the tumor, type of tumor, radiological findings (magnetic resonance imaging (MRI), computed tomography (CT)) scans, indication for surgery, diagnosis, clinical outcome, length of hospital stay (LOS), length of intensive care unit admission, the treatment proposed. Data on treatment modality, including surgery or conservative, were collected. Then the information was classified into continuous and categorized variables and analyzed accordingly.

### 2.3. Data Analysis

The research database is analyzed and processed using the statistical package for the social sciences (SPSS) software (version 23). The categorized variables were cross-tabulated using frequency tables and pie charts or bar charts. Chi-square test was used to obtain the significance of the association between categorized variables, using a *p*-value of ≤0.05 as the cut-off for significance. The numerical variables were summarized by their medians, means, and ranges, and the categorical variables were described by their counts and relative frequencies.

## 3. Results

Table 1 showing the demographic characteristics of the included cases in the present study. We have a total of 669 patients admitted to neurosurgical department at Khoula Hospital in Muscat—the capital city of the Sultanate of Oman—in a four-year period (from 2016 to 2019). Out of those 669 patients, 106 patients were admitted due to oncological pathologies, which will be the main focus of the present study. 58.5% of the study cohort were more than 75 years old. The maximum age was 89 and minimum age 65 with a mean age of 71.4 years.

The male-to-female ratio was (1:1.1). Meningiomas were the most commonly found type of tumors (52.8%) followed by GBM (18.9%). Most of the patients were having a GCS score of 14–15 on admission (69.9%). 35.8% of the patients received antiepileptic medications, most commonly phenytoin, during hospitalization. The vast majority of the patients underwent surgical intervention (68.9%); 63.2% of the patients stayed in the hospital less than 15 days.

Table 2 shows the association between the diagnosis of the patients and other variables (age, gender, GCS). It demonstrates that there was no significant difference between the different diagnosis categories (meningioma vs. non-meningioma) and the age of patients above and below 75 years. Also, it shows that there is no association between different diagnosis categories (meningioma vs. non-meningioma) and the patient’s gender across the study population (*p* = 0.336). However, it illustrates a significant difference among GCS scale (above and below 8) among patients with different types of tumors (meningioma vs. non-meningioma) (*p* < 0.05).

Table 3 shows the association between the LOS of patients and other variables (age and type of intervention). It illustrates that there was no significant relationship between the age of the patients (more and less than 75 years) and LOS (15 days as a cut-off value) (*p* = 0.939). Also, it demonstrates that there is a significant difference between LOS and the type of intervention (surgical vs. conservative). Patients with the conservative type of management were found to have a shorter LOS in the hospital compared with patients who underwent surgical intervention (*p* < 0.005).

**Figure 1:**
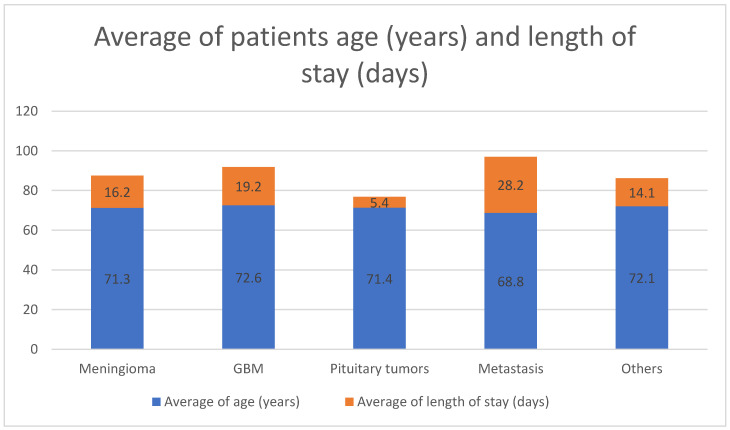
Average of patients’ age (years) and length of stay (days) in different types of tumors. GBM: glioblastoma multiform; Others: hemangioma, lymphoma, schwannoma.

Figure 1 represents the average age of the patients among the different types of tumors’, in which the GBM patients were found to have the highest mean age (72.6 years) followed by other tumors (hemangioma, lymphoma, schwannoma) tumors and pituitary tumors’ respectively (72.1 years, 71.4 years). It also demonstrates that patients with metastatic tumors had the longest length of stay in the hospital (mean of 28.2 days) followed by GBM patients (mean of 19.2 days).

## 4. Discussion

The incidence of CNS tumors increases with age, specifically, between the ages of 75 and 84. According to a study by Ramandeep S.Arora et al. the incidence of CNS tumor from the ages 25–84 is 14.57 per 100,000 person-years [19]. Meningiomas incidence after the age of 75 was 63.75 per 100,000 [7]. In our cohort more than half of the patients were more than or equal to 75 years of age. Meningioma cases after the age of 75 were higher compared to non-meningioma patients’ differences are evident in tumor incidence. According to the American Cancer Society, about 24,530 malignant tumors of the brain or spinal cord (13,840 in males and 10,690 in females) were diagnosed [20]. Reflecting on our cohort, this was contrary to our data, as more female patients were diagnosed with cancer compared to males. The commonest brain tumor in our cohort was meningioma which accounted for more than 50%. Meningioma occurred in a striking disparity, in which it was more than twice as likely to occur in adult females compared to males with an incidence of 8.44 per 100,000 persons [20]. Also, a study by Santosh et al. found that meningiomas are the most common tumor in the CNS. Brain tumors can be in a variety of locations in the brain. However, brain tumors commonly compress the base of the skull in the frontal lobe [21]. Correspondingly, our cohort found that the commonest site affected in the brain tumors was the frontal lobe. The vast majority of elderly patients had gliomas that were supratentorial compared to the younger population whereas infratentorial was common [22]. Reflecting on our cohort, supratentorial was the commonest site for brain tumors to tentorium cerebelli. In a study by Cohen-Inaber, the majority of patients diagnosed with meningioma had a GCS of 15 [23]. This runs parallel with the finding in our study as the majority of patients diagnosed with meningioma had GCS of more than 8 compared to non-meningioma patients with statistical significance. LOS was not dependent on age but on the type of treatment given to the patient and the presence of other comorbid conditions. However, the average LOS for patients diagnosed with meningioma was 18 days [23]. Nonetheless, when comparing the type of intervention to the LOS, we could see a statistical significance, as the LOS in surgical intervention is more than 15 days compared to conservative intervention which was almost 20 times different. This may be due to postoperative complications that might extend the LOS through management of the complications (e.g., reoperations, chest infections, urinary infections and other medical-related conditions), so providers may focus on preventing and managing complications to improve overall efficiency [24]. The increasing number of elderly patients with GBM corresponded to the increase in life expectancy. A study by Smrdel et al. found that the average age of patients diagnosed with GBM was 73 years of age [25]. Likewise, in our cohort, the average age of patients diagnosed with GBM was 72.6 years of age. Antiepileptic drugs (AEDs) are frequently prescribed peri-operatively in an attempt to reduce the risk of seizures post-craniotomy in meningioma patients [26]. Reflecting on our patients, only a minority of patients took AED as prophylaxis. Finally, patients with metastatic tumors had an extending LOS due to two factors; medical/surgical and socioeconomic [27]. Even though not observed in our study, we recognized that it is also important to study socioeconomic and medical/surgical factors determining LOS, similar to what is reflected in recent literature [27]. The medical/surgical factors can be further divided into preoperative, intraoperative and postoperative factors. An example of intraoperative factor is the number of instrumentation levels and postoperative examples are the complications that are found. On the other hand, the example of socioeconomic factors extending LOS is family support, and with better family support patient is more confident of being discharged resulting in shorter LOS [27].

## 5. Limitations

There were several limitations in this study. It was a retrospective, single-centered cross-sectional study over four years. Therefore, several confounding factors exist, such as the availability of diagnostic imaging facilities, advancement in modern medical technology, and improvement in the intensive care unit. Further studies in the future are recommended, keeping in mind the consideration of the current limitations. Follow-ups were not involved in this study.

## 6. Conclusions

Aging is considered one of the major risk factors for brain tumors. Locally, no previous studies have investigated the prevalence of geriatric neuro-oncology. The commonest CNS tumor in KH was meningioma followed by GBM. The number of geriatric neuro-oncology cases remained stable over the four years. Finally, the GCS score was higher in meningioma patients. Furthermore, special care must be taken when dealing with geriatric neuro-oncological cases due to the high potential rate of mortality and morbidity among those group, and a more holistic approach is recommended as an essential need to evaluate the overall situation of those patients and manage them accordingly.

## Figures and Tables

**Table 1:** Demographic characteristics of the included patients.

Category	Number of Patients (%)
Number of patients admitted each year	
2019	202 (30.0%)
2018	172 (25.7%)
2017	154 (23%)
2016	141 (21.3%)
Total number of admitted neurosurgical cases (2016–2019)	669
Total number of Neuro-oncological cases	106
Age	
≥75	62 (58.5%)
<75	44(41.5%)
Gender	
Female	54 (50.9%)
Male	52 (49.1%)
Type of tumors	
Meningioma	56 (52.8%)
GBM	20 (18.9%)
Pituitary tumors	7 (6.6%)
Metastatic cancer	11 (10.4%)
*Others	12 (11.3)
Anatomical locations of tumors	
Frontal	36 (34%)
Temporal	9 (8.5%)
Parietal	22 (20.8%)
Occipital	3 (2.8%)
Cerebellar	3 (2.8%)
More than one lobe	21 (19.8%)
Spinal cord	4 (3.8%)
Pituitary	7 (6.6%)
Anatomical location of tumors to tentorium cerebelli	
Supratentorial	100 (94.3%)
Infratentorial	3 (2.8%)
Spinal cord	2 (1.9%)
More than one location	1 (0.9%)
Glasgow Coma Scale (GCS) on arrival	
15–14	74 (69.9%)
13–12	12 (11.3%)
9–11	8 (7.5%)
≤8	12 (11.3%)
Antiepileptic	
Drugs	
Yes	38(35.8%)
No	68 (64.2%)
Type of interventions	
Surgical	73 (68.9%)
Conservative	33 (31.1%)
Length of stay (LOS)	
≤15 days	67 (63.2%)
>15 days	39 (36.8%)

GBM: glioblastoma multiform. *Others: hemangioma, lymphoma, schwannoma.

**Table 2:** The association between the diagnosis of the patients and other variables (age, gender, GCS).

		AGE		Gender		GCS	
		Age Less than 75	Age More than 75	Male	Female	GCS Less than 8	GCS More than 8
Diagnosis	Meningioma	20	36	25	31	3	53
	Non-meningioma	24	26	27	23	9	41
*p*-value		0.20		0.336		0.04	

**Table 3:** The association between the LOS of the patients and other variables (age and type of intervention).

Length of Stay (LOS)	Age		Type of Intervention	
	<75 Years	≥75 Years	Surgical	Conservative
LOS less than 15 days	28	39	36	31
LOS more than 15 days	16	23	37	2
*p*-value	0.939		<0.005	

## Data Availability

From medical records of patients from the “Al-Shifa Health Information System” of Ministry of Health in Sultanate of Oman used in Khoula Hospital.

## Abbreviations

Khoula Hospital (KH), Length of stay (LOS), Glasgow coma scale (GCS), GBM (Glioblastoma), Anti-epileptic drugs (AED), Central Nervous System (CNS), World Health Organization (WHO). Anaplastic astrocytoma (AA).
